# Nicotinamide n-Oxide Attenuates HSV-1-Induced Microglial Inflammation through Sirtuin-1/NF-κB Signaling

**DOI:** 10.3390/ijms232416085

**Published:** 2022-12-16

**Authors:** Xiaowei Song, Wenyan Cao, Zexu Wang, Feng Li, Ji Xiao, Qiongzhen Zeng, Yuan Wang, Shan Li, Cuifang Ye, Yifei Wang, Kai Zheng

**Affiliations:** 1 Institute of Biomedicine, College of Life Science and Technology, Jinan University, Guangzhou 510632, China; 2 Guangdong Province Key Laboratory of Bioengineering Medicine, Jinan University, Guangzhou 510632, China; 3 School of Pharmaceutical Sciences, Health Science Center, Shenzhen University, Shenzhen 518060, China

**Keywords:** HSV-1, microglia, inflammation, nicotinamide n-oxide, Sirtuin-1

## Abstract

HSV-1 is a typical neurotropic virus that infects the brain and causes keratitis, cold sores, and occasionally, acute herpes simplex encephalitis (HSE). The large amount of proinflammatory cytokines induced by HSV-1 infection is an important cause of neurotoxicity in the central nervous system (CNS). Microglia, as resident macrophages in CNS, are the first line of defense against neurotropic virus infection. Inhibiting the excessive production of inflammatory cytokines in overactivated microglia is a crucial strategy for the treatment of HSE. In the present study, we investigated the effect of nicotinamide n-oxide (NAMO), a metabolite mainly produced by gut microbe, on HSV-1-induced microglial inflammation and HSE. We found that NAMO significantly inhibits the production of cytokines induced by HSV-1 infection of microglia, such as IL-1β, IL-6, and TNF-α. In addition, NAMO promotes the transition of microglia from the pro-inflammatory M1 type to the anti-inflammatory M2 type. More detailed studies revealed that NAMO enhances the expression of Sirtuin-1 and its deacetylase enzymatic activity, which in turn deacetylates the p65 subunit to inhibit NF-κB signaling, resulting in reduced inflammatory response and ameliorated HSE pathology. Therefore, Sirtuin-1/NF-κB axis may be promising therapeutic targets against HSV-1 infection-related diseases including HSE.

## 1. Introduction

Herpes simplex virus type 1 (HSV-1) is a double-stranded DNA virus and the most common human neurotropic virus. An infection HSV-1 causes keratitis, cold sores, occasionally acute but severe herpes simplex encephalitis (HSE), and long-term central nervous system damage [1,2]. Notably, the mortality rate of HSE patients without appropriate antiviral treatment can reach 70% [3,4]. Nucleoside analogs such as acyclovir and famciclovir are clinically commonly used anti-HSV-1 drugs, which have obvious virus resistance and lack directly inhibitory effects on inflammatory response in immune compromised patients [3]. Of note, a HSV-1 neurotropic infection is also closely associated with development of neurodegenerative diseases such as Alzheimer’s disease and Parkinson’s disease [5,6]. Therefore, there is an urgent need to develop new drugs to mitigate HSV-1 infection and HSE symptoms.

Microglia are the first line of defense against neurotropic virus infection [7]. Under physiological conditions, microglia exist in a resting state, and activated microglia have two states, the pro-inflammatory M1 type and the anti-inflammatory M2 type [2]. The M1 state is characterized by the production and secretion of pro-inflammatory cytokines (IL-1β, IL-6, and TNF-α) and reactive oxygen species, whereas M2 microglia facilitate resolution of inflammation through anti-inflammatory factors (IL-10, TGF-β) [8,9,10]. Many viral infections interfere with microglia functions, including SARS-CoV-2, HIV, and herpesviruses [7,11,12]. Upon early infection of HSV-1, microglia are activated via cellular surface Toll-like receptors (TLRs), such as TLR1/2, or endosome TLRs, such as TLR9 and TLR3, and subsequently release inflammatory cytokines and type I IFNs to recruit innate immune cells and initiate antiviral immune responses [2,13]. However, persistent and overactivation of microglia at the late phase of HSV-1 lytic infection or latent infection continuously release inflammatory cytokines and trigger aggressive immune responses, resulting in detrimental effects on the CNS. In addition, the excessive inflammation by overactivated microglia causes neurotoxicity to other cell types, further contributing to CNS diseases [13]. Therefore, targeting microglial excessive activation and inflammatory response is an important strategy for the treatment of HSV-1-associated CNS diseases [14,15]. For instance, chlorogenic acid attenuated the inflammatory response of HSV-1-infected BV2 microglia via the TLR2/TLR9-Myd88 signaling pathway [16]. Kaempferol-3-O-rhamnoside reduced microglial apoptosis and the secretion of pro-inflammatory factors to attenuate HSV-1-induced inflammatory damage to the brain [17]. Therefore, any drugs that drive the polarization of microglia from M1 to M2 are potent and effective candidates to improve HSE.

**Figure 1:**
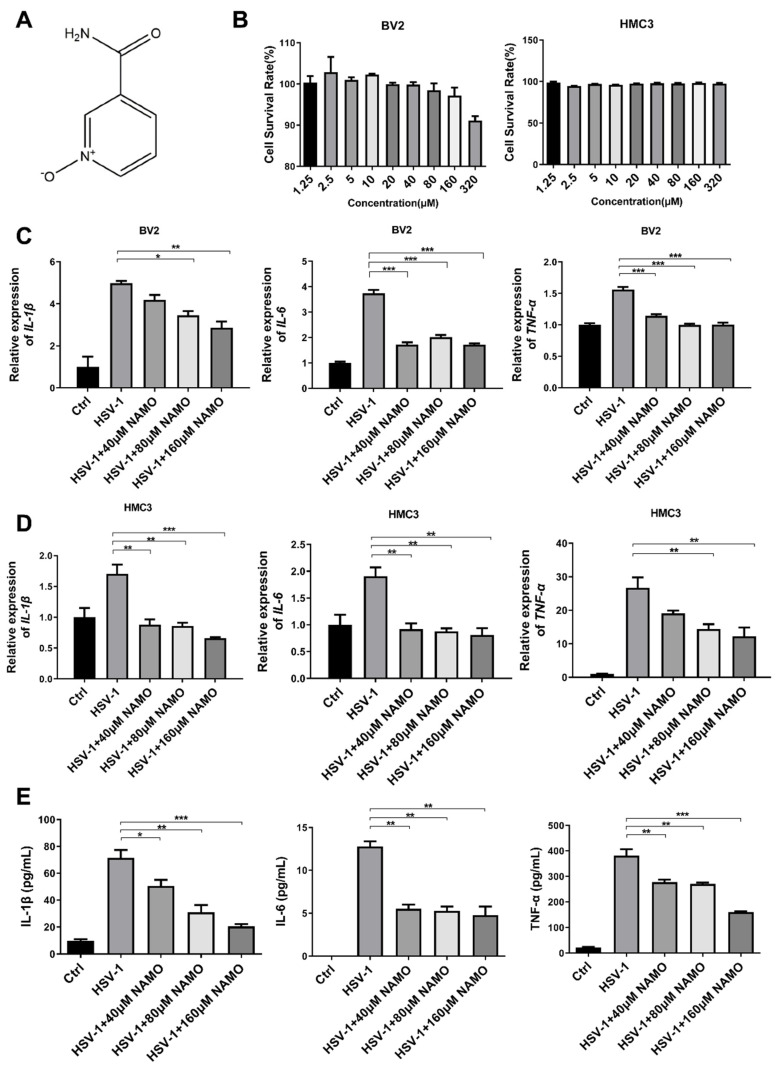
**NAMO inhibits the expression of HSV-1-induced cytokines**. (**A**) Chemical structure of NAMO. (**B**) BV2 and HMC3 cells seeded in 96-well plates were treated with different concentrations of NAMO for 48 h, and cell viability was then calculated by the MTT method. (**C**,**D**) BV2 and HMC3 cells were infected with HSV-1 (MOI = 1) in the presence of NAMO for 24 h, then the total RNA was extracted to detect the gene expression of *IL-1β*, *IL-6*, and *tnf-α* by RT-qPCR. (**E**) BV2 cells infected HSV-1 (MOI = 1) were treated with NAMO for 24 h, and the culture supernatant was subsequently collected for ELISA assay to detect the expression of HSV-1-induced cytokines. Data were presented as the means ± SEM (n = 3). * *p* < 0.05, ** *p* < 0.01, *** *p* < 0.001 vs. HSV-1-treated group, in an Unpaired *t* test.

Sirtuin-1 (SIRT1) is a member of the nicotinamide adenine dinucleotide (NAD)-dependent deacetylase Sirtuin family [18], and plays important roles in the regulation of aging, cell survival, differentiation and metabolism. SIRT1 regulates various key targets through deacetylation, such as histones, p53, NF-κB, PGC-1α and PPARγ [18,19,20]. Specifically, SIRT1 deacetylates the p65 subunit to inhibit NF-kB activity and suppresses inflammatory responses [21,22]. NAD+ precursors such as nicotinamide mononucleotide and nicotinamide riboside have also been demonstrated to promote SIRT1 activity to ameliorate neuroinflammation [23,24]. Nicotinamide n-oxide (NAMO) is an oxidative product derived from nicotinamide (Figure 1A) and our previous works have showed that NAMO, mainly produced by the gut microbe *Lactobacillus*, restricts the overactivation of microglia via the regulation of mitophagy to prevent or ameliorate HSE progression [14,15]. In this study, we explored a novel mechanism by which NAMO attenuates HSV-1-induced inflammation in microglia. We found that NAMO promotes microglial transition from the M1 to the M2 type, and enhances SIRT1 deacetylase enzymatic activity, which in turn inhibits NF-κB signaling through the deacetylation of the p65 subunit, leading to the reduction of pro-inflammatory cytokine production. Finally, we confirmed the anti-inflammatory effect and mechanism of NAMO in HSE mice.

## 2. Results

### 2.1. Cell Cytotoxicity and Anti-Inflammatory Activity of NAMO

The cytotoxicity of NAMO on BV2 (mouse microglial cells) and HMC3 (human microglial cells) was detected by a MTT assay, and the 50% cytotoxic concentrations (CC_50_) of NAMO on both microglial cells were >320 μM (Figure 1B). We then evaluated the effects of NAMO on HSV-induced cytokines at non-toxic concentrations. The mRNA expression levels of *IL-1β*, *IL-6*, and *tnf-α* were detected by RT-qPCR, which clearly showed that NAMO treatment reduced the expression of cytokines induced by HSV-1 infection on both cells (Figure 1C,D). Furthermore, the anti-inflammatory effect of NAMO was confirmed by ELISA assay (Figure 1E). The above data confirmed that NAMO reduced microglial inflammation triggered by HSV-1 infection.

### 2.2. NAMO Promotes the Microglia Transition from M1 to M2 Type

**Figure 2:**
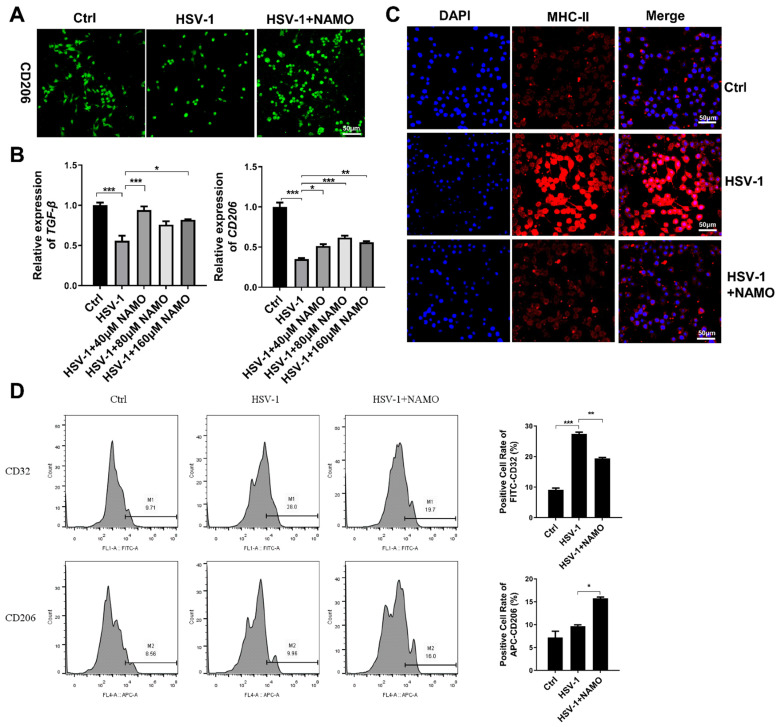
**NAMO switches microglial polarization from M1 to M2.** (**A**) BV2 cells were infected with HSV-1 (MOI = 5) with or without NAMO (160 μM) for 6 h. The cells were then fixed and stained with anti-CD206 antibody (green). Scale bar, 50 μm. (**B**) BV2 cells were infected with HSV-1 (MOI = 1) for 6 h in the presence or absence of NAMO, and the mRNA expression levels of M2 marker *TGF-β* and *CD206* were detected by RT-qPCR. (**C**) BV2 cells were infected with HSV-1 (MOI = 5) with or without NAMO (160 μM) for 6 h. The cells were then fixed and stained with anti-MHC-II antibody (red) and DAPI (blue). Scale bar, 50 μm. (**D**) BV2 cells were plated in 6-well plates at 4.0 × 10^5^ cells/well and co-treated with NAMO (160 μM) and HSV-1 (MOI = 5) for 6 h. The positive cells of CD32 (M1 marker) and CD206 (M2 marker) were detected by flow cytometry. Data were presented as the means ± SEM (n = 3). * *p* < 0.05, ** *p* < 0.01, *** *p* < 0.001 versus indicated group, Unpaired *t* test.

Next, we investigated whether NAMO treatment altered the phenotype of microglia when considering that microglial activation is heterogeneous and can be divided into a pro-inflammatory cytotoxic M1 phenotype and an alternative anti-inflammatory M2 phenotype with neuroprotective effects [8]. M2 microglia exert anti-inflammation resolution through anti-inflammatory factors (e.g., TGF-β, IL-10, IL-13) to reestablish homeostasis [10]. We firstly assessed the cellular morphological change of microglia via immunofluorescent staining with the microglial marker IBA-1. As shown in Supplemental Figure S1, HSV-1 infection triggered the transformation from resting microglia to a form with increased cytokine production, which was reversed by NAMO treatment and consistent with previous work [14]. We also analyzed the expression of M2-type activation marker CD206 via immunofluorescent staining and observed increased fluorescence of CD206 in HSV-1 infected cells by NAMO treatment (Figure 2A). In addition, through a RT-qPCR assay, we found that HSV-1 infection inhibited the mRNA expression of CD206 and M2-type associated cytokine TGF-β (Figure 2B), which were upregulated by NAMO treatment. Furthermore, an immunofluorescent assay indicated that HSV-1 largely enhanced the expression of M1-type marker MHC-II, whereas NAMO reduced its expression to a normal level (Figure 2C). Finally, we examined the ratio of M1 and M2 positive cells after stimulation with HSV-1 and NAMO by flow cytometry assay. As shown in Figure 2D, HSV-1 infection significantly upregulated the expression of M1-type marker, CD32. On the contrary, NAMO treatment decreased CD32 expression and promoted the expression of M2 marker CD206. Collectively, these results suggested that NAMO promotes the M1 to M2 transition of HSV-1-infected microglia.

### 2.3. NAMO Suppresses NF-κB Activation

**Figure 3:**
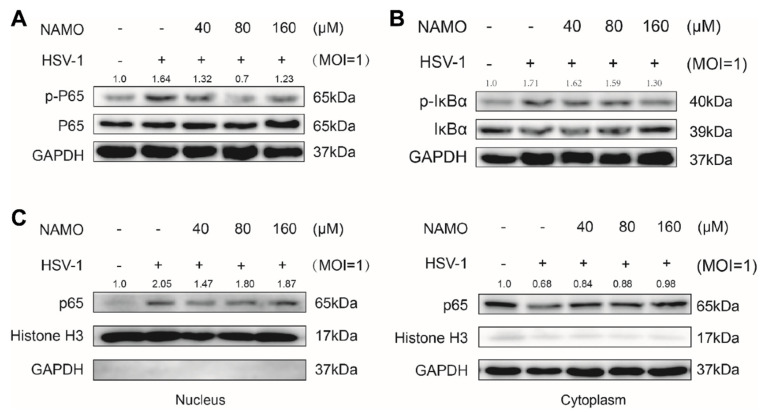
**NAMO suppresses NF-κB activation**. (**A**,**B**) BV2 cells infected with HSV-1 (MOI = 1) were treated with NAMO for 6 h, the protein levels of phorspho-NF-κB p65, NF-κB p65, p- IkBα, IkBα, and GAPDH were determined by western blotting. (**C**) All cytosol and nuclear proteins were extracted, and the expression level of p65 was detected.

Next, we assessed whether NAMO inhibited NF-κB signaling, the critical inflammation transducer, to reduce HSV-1-caused neuroinflammation. The protein levels of phosphorylated NF-κB p65 (p-p65), NF-κB p65, p-IkBα, and IkBα were detected by western blot assay. The result showed that p-NF-κB, p65, and p-IkBα were increased in HSV-1-treated BV2 cells, which were significantly decreased by NAMO treatment (Figure 3A,B). The NAMO-mediated inhibition of NF-κB signaling was further confirmed by detecting the nuclear translocation of p65. As shown in Figure 3C, NAMO significantly reduced nuclear p65 while increasing cytoplasmic p65. Consequently, the immunoblotting results indicated that NAMO inhibited NF-κB activation in HSV-1-infected BV2 cells.

### 2.4. NAMO Activates SIRT1 to Deacetylate p65 and Inhibit Inflammation

To further explore the specific mechanism of NAMO, we predicted the possible molecular targets of NAMO through several online prediction tools, including SwissTargetPrediction (http://swisstargetprediction.ch/ (accessed on 2 July 2019)), Super-PRED (https://prediction.charite.de/index.php?site=chemdoodle_search_target (accessed on 1 July 2008)), and SEA (https://sea.bkslab.org/ (accessed on 1 February 2007)). Notably, SIRT1 was one of the top candidates in all three predictive lists. In addition, NAMO analogues, nicotinamide mononucleotide and nicotinamide riboside have been demonstrated to promote SIRT1 activity [21,22]. In addition, SIRT1 suppressed inflammatory response through deacetylating p65 to inhibit NF-κB activity [20]. Therefore, we investigated the possible interaction between NAMO and SIRT1 by molecular docking. As shown in Supplemental Figure S2A,B, NAMO was well bounded in the activate substrate site of SIRT1 (PDB: 4ZZJ), interacting with several key resides (e.g., Pro-271) involved in the binding with the well-known SIRT1 activator, resveratrol. NAMO even exhibited a lower binding energy and docking score than that of resveratrol (Figure S2C), implying a stronger binding affinity between NAMO and SIRT1.

**Figure 4:**
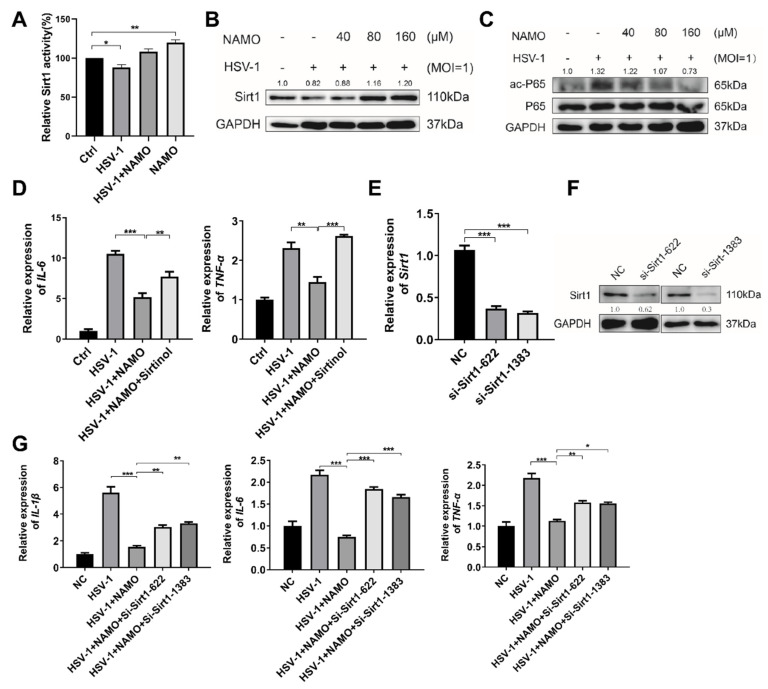
**NAMO inhibits NF-κB via SIRT1-mediated deacetylation of p65**. (**A**) BV2 cells infected with HSV-1 (MOI = 1) were treated with NAMO for 6 h, and the enzymatic activity of SIRT1 was analyzed. (**B**) BV2 cells were treated with NAMO for 6 h, and the protein level of SIRT1 was detected by western blotting. (**C**) Acetylated p65 (ac-p65) and total p65 were detected by western blotting. (**D**) BV2 cells were pretreated with 10 μM Sirtinol for 24 h, then infected with HSV-1 (MOI = 1) and co-treated with 160 μM NAMO for 6 h. Total RNA was extracted, and the mRNA expression levels of *IL-6* and *tnf-α* were detected by RT-qPCR. (**E**) BV2 cells were transfected with si-*Sirt1* for 24 h, the mRNA expression level of *Sirt1* was estimated by RT-qPCR. (**F**) BV2 cells were transfected with si-*Sirt1* for 48 h, and the protein level of SIRT1 was detected by western blotting. (**G**) BV2 cells were transfected with si-*Sirt1* for 24 h, then the cells were treated with NAMO for 6 h. Total RNA was extracted to detect the gene expression of *IL-1β*, *IL-6,* and *tnf-α* by RT-qPCR. Data were presented as the means ± SEM (n = 3). * *p* < 0.05, ** *p* < 0.01, *** *p* < 0.001 versus indicated group, Unpaired *t* test.

We then analyzed whether NAMO activated SIRT1 to inhibit NF-κB signaling and inflammation. We firstly found that NAMO significantly enhanced the deacetylase enzymatic activity of SIRT1 (Figure 4A). In addition, HSV-1 suppressed SIRT1 expression, whereas NAMO treatment increased the expression of SIRT1 (Figure 4B). NAMO also decreased the protein level of acetylated NF-κB p65 (Figure 4C), implying SIRT1-mediated deacetylation of p65 by NAMO. In addition, inhibition of SIRT1 by its potent inhibitor Sirtinol [25,26] attenuated NAMO-mediated anti-inflammatory effects, as evidenced by the restored expression levels of *IL-6* and *tnf-α* (Figure 4D). Furthermore, the *Sirt1* gene was knocked down by specific siRNAs, and the mRNA and protein levels of SIRT1 were confirmed (Figure 4E,F). Consistently, we found that siRNA-*Sirt1* impaired the anti-inflammatory effect of NAMO (Figure 4G). Taken together, these results suggest that NAMO suppresses inflammatory response by activating SIRT1 to promote NF-κB p65 deacetylation.

### 2.5. NAMO Activates SIRT1 to Ameliorate HSE Pathology In Vivo

**Figure 5:**
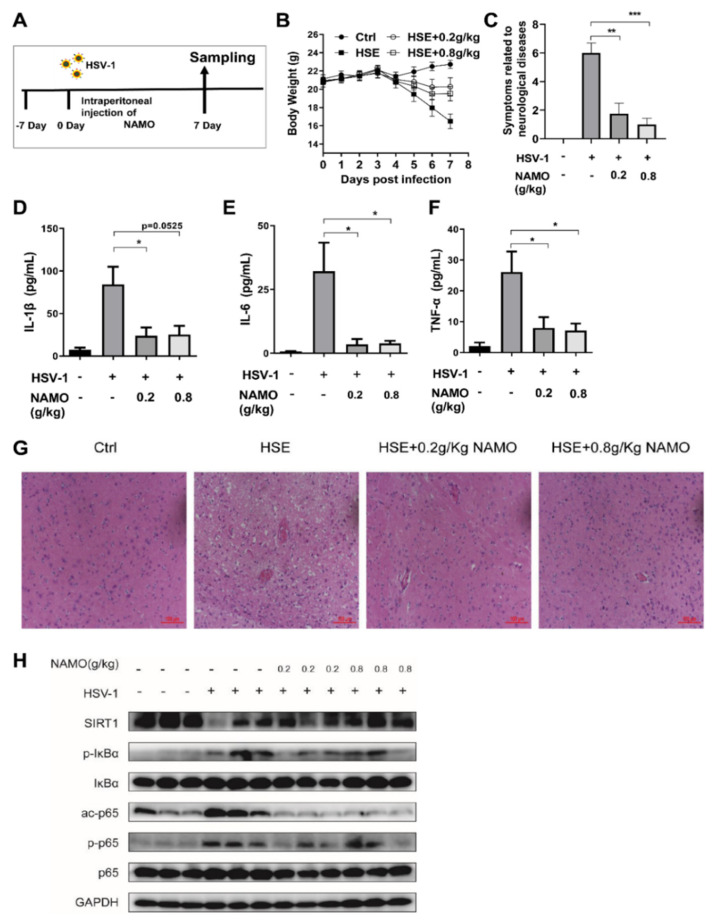
**NAMO activates SIRT1 to mitigate HSE in vivo** (**A**) Schematic protocol for HSE mice. (**B**) The body weight of mice was recorded daily (n = 8). (**C**) NAMO improves neurological disease score related HSE symptoms (n = 8). Different neurodegenerative symptoms were scored and summed. (**D**–**F**) ELISA analysis of cytokine production. Serum cytokine IL-1β (**D**), IL-6 (**E**), and TNF-α (**F**) in HSV-1 infected mice at 7 days post infection were detected (n = 5). Data were presented as the means ± SEM. * *p* < 0.05, ** *p* < 0.01, *** *p* < 0.001 versus indicated group, Unpaired *t* test. (**G**) Cerebral cortex tissues derived from HSE mice (n = 3) were subjected to H&E staining and representative images were shown. Scale bar, 100 μm. (**H**) Immunoblot analysis of SIRT1/NF-κB signaling pathway with the indicated antibodies from mouse brain tissue (n = 3).

Finally, we investigated whether NAMO suppresses HSV-1 infection and neuroinflammation via SIRT1 in vivo. A HSE mouse model was established as described before (Figure 5A), and HSE-associated weight loss and neurodegenerative symptoms were recorded. As expected and consistent with previous work [14], NAMO treatment reduced the weight loss (Figure 5B) and neurological disease scores of HSE mice (Figure 5C). In addition, we confirmed the anti-inflammatory effect of NAMO on the production of IL-1β, IL-6, and TNF-α in vivo by ELISA assay (Figure 5D–F). Furthermore, brain tissues of HSE mice were analyzed by H&E staining, which showed that NAMO significantly mitigated the HSV-1-induced neuroinflammation, as evidenced by the reduced number of damaged neurons, neuronal necrosis, cytoplasmic vacuolization, and lymphocytic infiltration (Figure 5G). Finally, western blot assay demonstrated that NAMO inhibited the protein levels of phosphorylated p65 (p-p65) and phosphorylated IkBα (p-IkBα) (Figure 5H). Specifically, NAMO promoted SIRT1 expression and reduced the expression of acetyl-Nf-κB p65 induced by HSV-1 infection, further supporting that NAMO inhibits neuroinflammation and HSE pathology via SIRT1-mediated deacetylation of p65.

## 3. Discussion

Reducing excessive microglial inflammation induced by HSV-1 infection is an effective strategy to alleviate HSE [2,27,28]. Herein, we demonstrated that gut microbial metabolite NAMO promotes microglial transition from the M1 to the M2 type and activates SIRT1-mediated deacetylation of p65 to inhibit microglial neuroinflammation and to reduce HSE pathology. HSV-1 infection has been shown to activate the NF-κB signaling pathway [29,30], which requires the acetylation of p65 [31]. Notably, by deacetylating p65, the NAD+ dependent deacetylase SIRT1 regulates inflammatory response in CNS diseases to multiple stimuli [32,33]. For example, asiatic acid suppresses neuroinflammation in BV2 microglia via modulation of the SIRT1/NF-κB signaling pathway [22]. Therefore, the SIRT1/NF-κB axis represents an attractive anti-neuroinflammation target against HSV-1 infection. It is also confirmed in our work that HSV-1-induced microglia inflammation partially depends on the acetylation level of p65.

Microglial homeostasis plays critical roles in the immune response to different stimulus and CNS diseases. NAMO is a derivative of nicotinamide nitrogen oxides, and its analogue, nicotinamide mononucleotide, has been widely reported to have many biological functions, such as anti-inflammation, anti-infection, anti-oxidation, antipruritic, and anticancer [34,35]. Our previous study demonstrated that NAMO, mainly produced by gut microbes *Lactobacillus*, activates microglial mitophagy to reduce the production of inflammatory cytokines, and inhibits HSV-1 replication in neuronal cells [14,15]. However, the mechanism by which NAMO activates mitophagy remains to be determined. In this study, we identified a novel mechanism, in which NAMO promotes SIRT1 deacetylase activity and increases SIRT1 expression to inhibit inflammation (Figure 4A,B). These observations were further confirmed by the application of SIRT1 inhibitor Sirtinol, pretreatment with which attenuated the anti-inflammatory effects of NAMO. Notably, SIRT1 has also been extensively demonstrated to activate mitophagy [36,37,38], implying that NAMO suppresses microglial inflammation, through at least two possible paracrine signaling pathways, the SIRT1-mitophagy and the SIRT1/NF-κB axis. Furthermore, restricting the activation of microglia by microglial inhibitors, such as the complement C3a receptor (C3aR) antagonist SB290157, has been demonstrated to limit neuroinflammation and consequently neuronal death [39]. It is therefore interesting to test whether NAMO modulates C3aR activity to reduce microglial inflammation, or whether SB290157 has similar antiviral activity against HSV-1 infection and HSE pathology.

Another finding in our work is the involvement of SIRT1 in HSV-1-induced excessive microglial inflammation. SIRT1 exerts a neuroprotective function in age-related neurodegenerative diseases and several clinical trials have been performed to investigate the beneficial effect of SIRT1 activator resveratrol in neurodegenerative patients [40]. To date, only a few studies have demonstrated the interaction between HSV-1 infection and SIRT1 activation, as evidenced by the antiviral activity of resveratrol [41,42,43]. For instance, Leyton et al. demonstrated that HSV-1 infection modulates SIRT1 activity to regulate mitochondrial biogenesis and apoptosis of neuronal cells, whereas activation of SIRT1 increases neuronal cell viability, inhibits HSV-1 gene expression, and reduces neurodegenerative biomarkers induced by HSV-1 in neuronal cells [43]. Herein, we revealed that microbial metabolite NAMO acts as a potential SIRT1 activator, which stimulates SIRT1 to restrict microglial excessive inflammation in vitro and mitigate HSE pathology in vivo. Considering that NAMO is mainly produced by the gut microbes *Lactobacillus* [14], the anti-inflammatory effect of NAMO further implicates the possible contribution of the microbiota–gut–brain axis to SIRT1-mediated protection [40]. Several works have indeed established the interaction between gut microbe *Lactobacillus* and SIRT1 activity in different diseases such as aging [44], colitis [45], sepsis [46], and anxiety [47].

Finally, the SIRT1/NF-κB axis has also been shown to regulate microglial polarization [48]. In fact, our previous work clearly implicated that HSV-1 infection causes the microglial M2 to M1 transition [14,15]. By RNA-sequencing, we found that HSV-1 enhances M1 marker expression and reduces M2 marker expression in both brain tissues and primary isolated microglia. In addition, we showed that NAMO treatment reduces the number of overactivated microglia in different brain regions of HSE mice. Our present work strengthened this hypothesis and further works are required to determine how NAMO-SIRT1 activation promotes the microglial M1 to M2 transition.

In summary, our study elucidated the role of gut microbial metabolite NAMO in activating SIRT1, which in turn deacetylates p65 to inhibit NF-κB signaling and HSE-associated neuroinflammation. Therefore, NAMO may act as an SIRT1 activator with beneficial effects on herpesvirus encephalitis.

## 4. Materials and Methods

### 4.1. Cells, Virus and Regents

Mouse microglia BV2 cells were purchased from the National Infrastructure of Cell Line Resource in China (3111C0001CCC000063, Shanghai, China). Human microglia HMC3 cells (CRL-3304) and African green monkey kidney epithelial Vero cells (CCL81) were purchased from American Type Culture Collection (ATCC, Manassas, VA, USA). Cells were cultured in DMEM (8118305, GIBCO, Carlsbad, CA, USA) supplemented with 10% fetal bovine serum (FND500, ExCell Bio, Shanghai, China). HSV-1 strain F was obtained and stored as previously indicated [14].

NAMO (S4785) and Sirtinol (S2804) was purchased from Selleck Chemicals (S4785, Houston, TX, USA). The enzyme-linked immunosorbent assay (ELISA) kit of IL-1β (CME0015), IL-6 (CME0006) and TNF-α (CME0004) were purchased from (Sizhengbai, Beijing, China). The cell SIRT1 colorimetry assay kit (GMS 50287.1) was purchased from GENMED (GENMED Scientifics Inc., Shanghai, China). Antibodies including anti-GAPDH (5174S), anti-IBA-1 (17198), anti-SIRT1 (9475), anti-P-p65 (3033S), and anti-p65 (8242S) were obtained from Cell Signaling Technology (Danvers, MA, USA). Anti-NF-kB p65 (ab19870), anti-CD32 (ab282740) and anti-MHC-II (ab25333) were provided by Abcam (Cambridge, UK). IκB (sc-1643) and p-IκB (sc-8404) were obtained from Santa Cruz Biotechnology (Santa Cruz, CA, USA). CD206 (187041-AP) antibody was purchased from Proteintech (Proteintech Group Inc., Chicago, IL, USA). TRIzol Reagent was bought from TIANGEN (DP405, Beijing, China).

### 4.2. MTT Assay

The cytotoxicity of NAMO on BV2 and HMC3 cells was assessed via a MTT assay. Both BV2 and HMC3 cells were seeded in 96-well plates in 100 μL growth media, cultured for 24 h, followed by NAMO treatment at various concentrations for 24 h. Then, 10 μL of MTT solution (5 mg/mL) was added to each well and incubated at 37 °C for another 2 h. The absorbance was then measured at 570 nm using an enzyme immunoassay reader (Bio-Rad, Hercules, CA, USA).

### 4.3. Real-Time Quantitative PCR (RT-qPCR)

BV2 and HMC3 cells were infected with HSV-1 (MOI = 1) in the presence or absence of NAMO. Total RNA was isolated from cultured cells using TRIzol reagent (DP405, Beijing, China) and were then reversely transcribed into cDNA with a PrimeScript RT kit (RR036A-1, TaKaRa, Shiga, Japan). The relative expression of mRNA was determined by using TB Green ^®^ Premix Ex Taq™II (RR820A, TaKaRa, Shiga, Japan) with the CFX96 Touch™ Real-Time PCR Detection System (Bio-Rad, Hercules, CA, USA). Data were processed and analyzed using the Bio-Rad CFX Manager software. The mRNA expression levels were normalized to the internal housekeeping gene *gapdh*. The gene-specific primers are showed in Table 1.

### 4.4. Enzyme-Linked Immunosorbent Assay (ELISA)

BV2 cells were infected with HSV-1 for 24 h in the presence or absence of NAMO, and the cell supernatant was then collected. In addition, mouse blood was collected, coagulated for 30 min at room temperature, centrifuged at 1000× *g* for 10 min, and the serum was carefully separated. Next, the levels of IL-1β (CME0015), IL-6 (CME0006), and TNF-α (CME0004) was evaluated by an ELISA kit (Sizhengbai, Beijing, China) according to the corresponding manual.

### 4.5. Western Blot Assay

BV2 cells were infected with HSV-1 in the presence or absence of NAMO. Cell lysates were collected using RIPA Lysis Buffer (P0013B, Beyotime, Shanghai, China) with 1% PMSFS (T506, Beyotime, Shanghai, China). Then protein concentrations were determined with a BCA protein assay kit (P0009, Beyotime, Shanghai, China). A protein extraction kit was used to disassociate nuclear and cytoplasmic proteins according to the manufacturer’s protocol (P0027, Beyotime, Shanghai, China). Proteins were separated by 8–12% gradient SDS-PAGE, transferred to polyvinylidene fluoride membranes (Millipore, Darmstadt, Germany), and blocked with 5% nonfat milk for 1 h at room temperature. The corresponding primary antibodies were incubated overnight at 4 °C, followed by incubation with the appropriate horseradish peroxidase conjugated secondary antibodies for 1.5 h at room temperature. Finally, these target proteins were detected with ECL solution and imaged with a 5200-image analysis system (Tannon, Shanghai, China). The band intensity was quantified by the ImageJ software (Bio-Rad, Hercules, CA, USA). Band intensities for each protein were normalized to GAPDH.

### 4.6. Detection of Enzymatic Activity of SIRT1

BV2 cells were plated in 100 mm dishes at a density of 1 × 10^7^ per well and incubated for 24 h. Then the cells infected with or without HSV-1 (MOI = 1) were treated with NAMO (160 µM) for 6 h. Next, each group of cells was collected to detect the deacetylase enzymatic activity of SIRT1 according to the instruction manual of the SIRT1 colorimetry assay kit (GMS 50287.1) from GENMED (GENMED Scientifics Inc., Shanghai, China).

### 4.7. Flow Cytometry

BV2 cells were plated in 6-well dishes at a density of 4 × 10^5^ per well and incubated for 24 h. Then the cells infected with or without HSV-1 (MOI = 5) were treated with NAMO (160 µM) for 6 h. After fixation, the cells were directly incubated with primary anti-CD206 or anti-CD32 antibody at 4 °C overnight, respectively. The cells were then incubated with FITC-conjugated or APC-conjugated antibody (406001/141707, BioLegend, San Diego, CA, USA) at room temperature for 1 h. After washing three times with PBS, the cells were resuspended in 300 μL of 1× PBS solution. Finally, the fluorescence intensity was analyzed with a flow cytometer (LSR II, Becton Dickinson, San Jose, CA, USA).

### 4.8. siRNA Transfection

All siRNAs were synthesized by GenePharma Co, Ltd. (Shanghai, China) and transfected using INTERFER in reagent (PT-409-10, Polypus Transfection, Illkirch, France) according to the kit’s instructions. Briefly, the siRNA was used at a non-toxic concentration of 100 nM. BV2 cells transfected with *Sirt1* siRNAs for 24 h were infected with HSV-1 (MOI = 1) for 24 h in the presence or absence of NAMO. All siRNA sequences were as follows: *Sirt1*-mus-622, GCACCGAUCCUCGAACAAUTT; *Sirt1*-mus-1383, GCACUAAUUCCAAGUUCUATT.

### 4.9. HSE Mice Model

All procedures for mouse studies were approved by the Jinan University Institutional Animal Care and Use Committee (No. 20200402-07). HSE mice were established as previously described [14,49]. Briefly, all male mice, purchased from Guangdong medical laboratory animal center at 4 weeks of age, were randomly divided into several groups. Mice were either treated with an equal volume of medium (mock-infected) or infected with 2 × 10^6^ PFU HSV-1 in a total of 20 μL of volume by a nasal intubation drip. The NAMO treatment group was administered by intraperitoneal injection of different concentrations every other day until the end of the experiment. All mice were weighed and disease scored with reference to the previous study [14,49]. Briefly, different HSE symptoms related to neurological disease were scored, including eye lesions (0: normal, 1: minor swelling, 2: moderate swelling, 3: severe swelling and skin lesions or lesions extensive), stimulus response (0: normal, 1: jumpy, 2: moderate response, 3: no movement or unresponsive), and crawl gait (0: normal, 1: uncoordinated, 2: hunched/lethargic, 3: Inability to walk spontaneously, loss of consciousness). Then, all scores were summed. Mice were sacrificed when weight loss reached approximately 20%.

### 4.10. Histology

The brain tissues of mice were collected, fixed with 4% paraformaldehyde, and sequentially embedded in paraffin and sectioned as previously described [14]. For hematoxylin-eosin (H&E) staining, the samples were deparaffinized and stained with H&E. The brain slices were then detected and analyzed with a light microscope.

### 4.11. Immunofluorescence Assay

Microglia M1 and M2 markers were analyzed by immunofluorescence assay as previously described [14]. Briefly, BV2 cells seeded in confocal dishes were treated with HSV-1 and NAMO for 6 h and the cells were then fixed with 4% paraformaldehyde, permeabilized with 0.1% Triton X-100, and blocked with 5% bovine serum albumin. Then, the cells were incubated with anti-CD206, anti-MHC-II, or anti-IBA-1 antibody, respectively, overnight at 4°C. Samples were incubated with Alexa Fluor labelled appropriate secondary antibody (A32790/A10239, Thermo Fisher Scientific, Waltham, MA, USA) for 1 h at room temperature. Subsequently, the nuclei were stained with DAPI (C1006, Beyotime, Shanghai, China). Finally, fluorescent images were obtained by a confocal laser scan microscope (LSM 510 meta, Zeiss, Germany).

### 4.12. Statistical Analysis

Data were presented as mean ± SEM. Data were analyzed by one-way analysis of variance or Student’s *t* test as appropriate, with p values less than 0.05 being considered statistically significant. n.s, not significant, * *p* < 0.05, ** *p* < 0.01 or *** *p* < 0.001.

## Figures and Tables

**Table 1:** The primer sequences for RT-qPCR.

Gene	Forward (5′-3′ Sequence)	Reverse (5′-3′ Sequence)
*IL-1β* (mouse)	AATGCCACCTTTTGACAGTGATG	AGCTTCTCCACAGCCACAAT
*IL-6* (mouse)	CTCCCAACAGACCTGTCTATAC	CCATTGCACAACTCTTTTCTCA
*tnf-α* (mouse)	AGGGTCTGGGCCATAGAACT	CCACCACGCTCTTCTGTCTAC
*gapdh* (mouse)	TGTGTCCGTCGTGGATCTGA	CCTGCTTCACCACCTTCTTGA
*IL-1β* (human)	GCCAGTGAAATGATGGCTTATT	AGGAGCACTTCATCTGTTTAGG
*IL-6* (human)	CACTGGTCTTTTGGAGTTTGAG	GGACTTTTGTACTCATCTGCAC
*tnf-α* (human)	GGGCCTGTACCTCATCTACT	TGACCTTGGTCTGGTAGGAG
*gapdh* (human)	AGCCTCAAGATCAGCAATG	CACGATACCAAAGTTGTCATGGAT

## Data Availability

The data presented in the study are included in the article. Further inquiries can be directed to the corresponding authors.

## Supplementary Materials

The following supporting information can be downloaded at: https://www.mdpi.com/article/10.3390/ijms232416085/s1.
