# Supplementary material for: Nicotinamide n-Oxide Attenuates HSV-1-Induced Microglial Inflammation through Sirtuin-1/NF-κB Signaling
Source: Int J Mol Sci. 2022 Dec 16;23(24):16085. doi: 10.3390/ijms232416085 (PMC9784159; doi:10.3390/ijms232416085)
Supplement: Supplementary file 1 [file ijms-23-16085-s001.zip › ijms-2012470-supplementary.pdf]

Supplemental information

Nicotinamide n-oxide attenuates HSV-1-induced microglial inflammation through Sirtuin-1/NF-κB signaling

Supplemental figure and legends

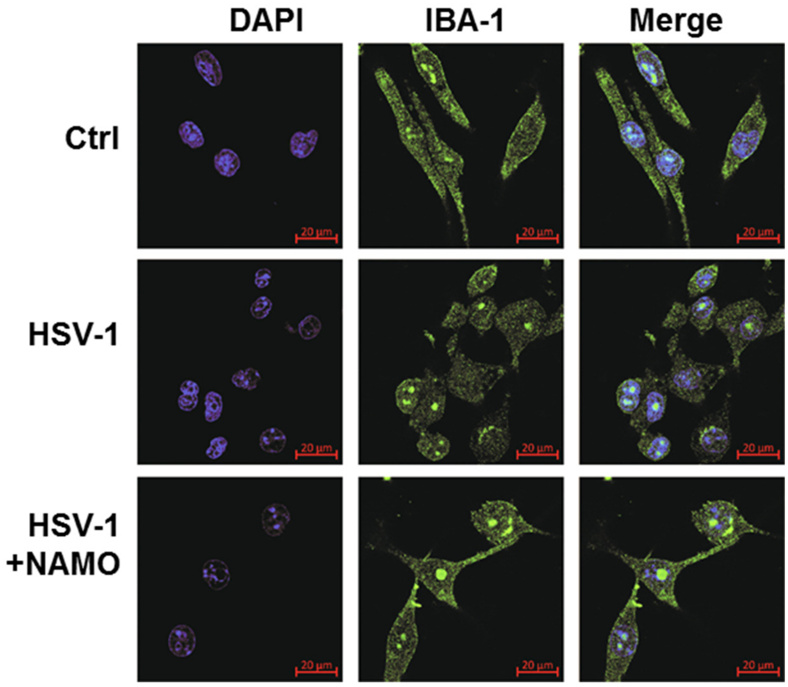

Figure S1. NAMO restores the morphology of microglia. BV2 cells were infected with HSV-1 (MOI=5) with or without NAMO (160 μM) for 6 h. The cells were then fixed, stained with anti-IBA-1 antibody (green) and DAPI (blue). Scale bar, 100 μm.

A

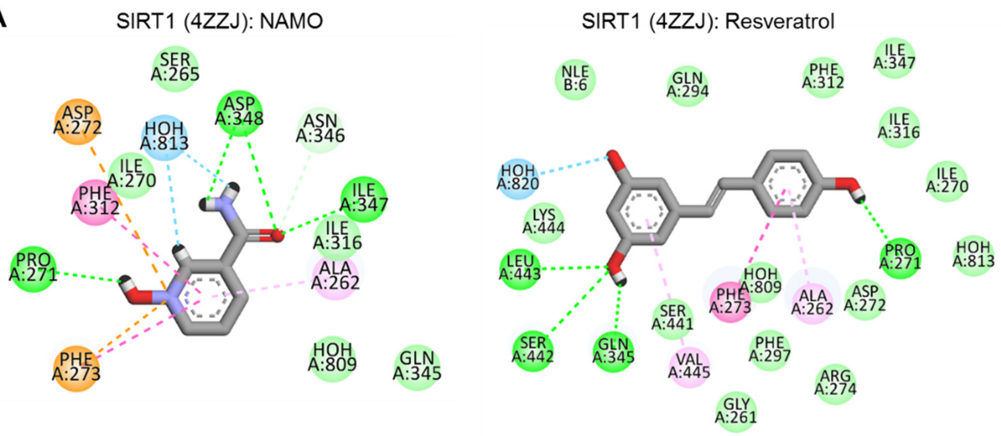

B

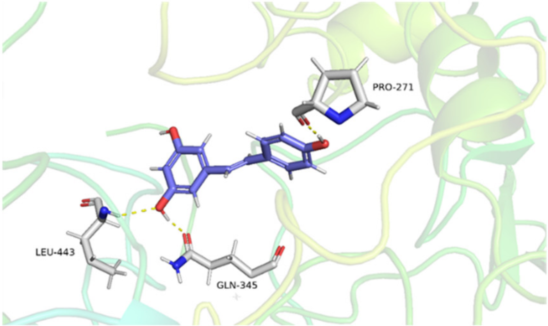

C

	MMGBSA dG Bind	Docking score
NAMO	-62.56	-9.302
Resveratrol	-42.99	-7.173

Figure S2. NAMO interacts with Sirtuin-1 by molecular docking. (**A** and **B**) 3D schematic diagram of the binding of NAMO to Sirtuin-1 (PDB: 4ZZJ). Sirtuin-1 was docked with NAMO or its activator resveratrol. (**C**) Molecular docking free binding energy and docking fraction.
